# Study on effectiveness analysis of multi-media combined with PBL teaching in breast surgery

**DOI:** 10.4314/ahs.v23i2.36

**Published:** 2023-06

**Authors:** Xusheng Zhang, Yiqing Guo, Ji Wang, Xudong Zhang, Junsheng Cheng, Haoqiang Chen, Qixing Pi

**Affiliations:** 1 General Surgery in Northwest Minzu University, Lanzhou 730000, Gansu Province, China; 2 ShiJiaZhuang Medical College, Shijiazhuang 050599, Hebei Province, China; 3 Northwest Minzu University, Lanzhou 730000, Gansu Province, China

**Keywords:** Multi-media, PBL, clinical teaching, breast surgery

## Abstract

**Objective:**

To explore the effectiveness of multi-media combined with PBL teaching in breast surgery.

**Methods:**

56 interns who came to our hospital for breast surgery from January 2019 to December 2020 were randomly divided into two groups: traditional teaching control group (n = 28) and multi-media PBL teaching observation group (n = 28). Two groups' theoretical knowledge, skill operation, teaching quality, student's evaluation and satisfaction with the teaching model were compared before ending the internship.

**Results:**

The total scores of breast examination, differential diagnosis, imaging reading, diagnosis and treatment scheme and total scores of the students in the observation group were higher than those of the students in the control group, and the total scores of body position, surgical site selection, disinfection and local anesthesia, surgical operation, postoperative treatment and clinical operation skills were also higher than those of the students in the control group (P < 0.05), but there was no significant difference between the two groups in medical history collection, professional knowledge and preoperative preparation (P > 0.05). The teaching quality of the students in the observation group was also significantly higher than that in the control group (P < 0.05). In addition, the students' satisfaction with the teaching method, the teaching effect and the overall satisfaction in the observation group were significantly higher than those in the control group (P < 0.05).

**Conclusion:**

Multi-media combined with PBL teaching can effectively improve students' professional knowledge theory level, operation skills, enhance students' enthusiasm and initiative, develop good clinical thinking habits, and have high teaching satisfaction. It is worthy of being popularized in the clinical teaching of breast surgery.

## Introduction

Breast surgery is a special clinical discipline, which has its own independent clinical characteristics and self-made diagnosis and treatment methods 1,2. However, most of its patients are female, and the disease sites are relatively private. In addition, in the teaching syllabus of breast surgery, there are less lectures and more contents, which brings various challenges to clinical teaching 3. In the course of graduate student's internship, the traditional teaching model with the focus on theory teaching has some discontinuity between theory and practice, which is easy to lead to the loss of students' interest in study and the poor effect of internship 4. Problem Based Learning (PBL), which is guided by problems and led by teaching with student-oriented, has been gradually developed in medical education and achieved good teaching results 5,6. With the development of medical technical level and the improvement of people's requirement for medical service, the implementation of diversified teaching model is also advocated in the course of education and teaching of breast surgery. Because of its flexible and changeable characteristics, multi-media teaching has broken the old dull teaching mode 7. Students can learn selectively according to their own needs, and enhance the interaction of teaching and improve the level of teaching 8. In order to improve the clinical teaching quality of clinical medical students and strengthen the clinical teaching management of breast surgery, we combine multi-media teaching with PBL teaching to explore the effective value of this new clinical teaching model in breast surgery.

## Data and Methods

### General data

56 interns who came to our hospital for breast surgery from January 2019 to December 2020 were divided into the control group and the observation group with digital random method. Each group consisted of 28 interns, aged 22-26 years. The average age was (23.43 ± 3.12) years. The ratio of male to female was 4:3. There was no difference between the two groups in general data (P > 0.05).

### Research method

In the control group, the traditional teaching model was adopted. The teacher explained the professional knowledge related to the mammary gland, introduced the management system and workflow in the hospital, and led the students to understand the operation specifications of ward environment, demonstration, consultation, dressing change and surgical diagnosis and treatment.

The observation group adopts the multi-media teaching combined with PBL teaching model, and the concrete operations are as follows:

(1). The teacher draws up the PBL teaching plan in advance according to the syllabus and the situation of the patients admitted to the ward of the hospital, puts forward some clinical problems before the implementation of the teaching plan, and finally prints out the plan and distributes it to the interns of the observation group.

(2). The interns of the observation group make preparations after getting the outline of the PBL teaching plan, the responsible person divides the interns into the study group 30 minutes before the teaching to carry on the discussion and the analysis to the pre-class questions, to obtain the answers, and to carry on the summary and the analysis to the answers.

(3). The instructor uses the multi-media courseware to carry on the professional teaching, through the operation video and the picture and soon, it makes the student master the theory key points; after the course, the students need each to express oneself and the free discussion to solve the problems that the teacher left behind.

(4). The instructor leads the interns to the ward for practical study, including questioning of medical history, physical examination and relevant laboratory examination results. After the end of the course, review the teaching video, consolidate and strengthen the classroom knowledge, use the test questions behind the video to consolidate the knowledge points. After the course, the interns also need to think about the diagnosis results of the patients and the relevant differential diagnosis, and give the treatment plan according to the actual situation of the patients.

(5) After the course, each group will discuss the medical records in combination with the ward round content, and give the discussion contents to the instructor, who will correct and give feedback.

### Observation indicators

(1). To compare the results of theoretical knowledge assessment of the two groups, the total score is 50 points, including professional knowledge, medical history collection, breast examination, imaging reading, differential diagnosis, and diagnosis and treatment scheme.

(2). To compare the scores of operational assessment of the two groups, the total score is 50 points, including preoperative preparation, disinfection and local anesthesia, body position and surgical site selection, surgical operation and postoperative treatment.

(3). To compare whether the teaching quality of the two groups is improved or not.

(4). To compare two groups' evaluation of teaching model, such as teaching attitude, teaching content, teaching method and teaching effect.

(5). To compare two groups' satisfaction with teaching, overall satisfaction = (very satisfied + satisfied + basically satisfied) / number of cases × 100%.

### Statistical analysis

SPSS 26.0 software was used for statistical analysis of the data in this study. Comparison of measurement data between two groups used t test method, expressed by (^-^ x ± s); count data used χ^2^ test method, expressed by rate (%). P<0.05 indicates a difference, that is, the results are statistically significant.

## Result

### Theoretical knowledge evaluation results of the two groups

There was no significant difference between the students in the control group and the observation group in terms of professional knowledge and medical history collection, and there were no obvious advantages and disadvantages (P > 0.05). However, the students in the control group were significantly higher than that in the control group in terms of breast examination, differential diagnosis, imaging reading and diagnosis and treatment scheme (P < 0.05). The total score of the observation group was 44.17 points, which was higher than that of the control group (39.71 points) (P < 0.05). See Table 1 for details.

**Table 1:** Comparison of Test Results of Theoretical Knowledge between the Two Groups (points, x̅ ± s)

Group	Number of cases	Professional knowledge	Medical history collection	Breast examination	Differential diagnosis	Imaging reading	Diagnosis and treatment scheme	Total score
Control group	28	24.11±2.43	4.17±1.54	3.43±0.52	3.11±0.59	3.44±0.51	3.51±0.78	39.71±1.53
Observation group	28	24.32±2.32	4.21±1.62	4.30±0.48	4.22±0.61	4.29±0.48	4.31±0.64	44.17±2.61

t value		-0.331	-0.095	-6.505	-6.921	-6.422	-4.196	-7.801
P value		0.742	0.925	0.000	0.000	0.000	0.000	0.000

### Operation evaluation scores of the two groups

The scores of body position, surgical site selection, disinfection and local anesthesia, surgical operation and postoperative treatment in the observation group were significantly higher than those in the control group, and the differences were significant (P < 0.05); however, there was no difference in preoperative preparation between the two groups (P>0.05); the total score of the observation group was 48.51 points, which was significantly higher than that of the control group (44.03 points, P < 0.05), as shown in Table 2.

**Table 2:** Comparison of the Scores of Operation Examination between the Two Groups (points, x̅ ± s)

Group	Number of cases	Preoperative preparation	Body position and surgical site selection	Disinfection and local anesthesia	Surgical operation	Postoperative treatment	Total score
Control group	28	4.33±0.71	7.18±0.87	7.86±1.12	14.76±0.87	3.88±0.67	44.03±3.55
Observation group	28	4.54±0.62	9.38±0.74	9.45±0.94	18.96±1.00	4.71±0.82	48.51±2.84

t value		-1.179	-10.192	-5.754	-16.767	-4.148	-5.214
P value		0.244	0.000	0.000	0.000	0.000	0.000

### Teaching quality of the two groups

The results of the questionnaire showed that among the students in the observation group, 27 improved their learning interest, 26 improved their problem-solving ability and clinical thinking, and 25 improved their autonomous learning ability, and the number of students were all significantly greater than that of the control group. The difference was significant (P < 0.05). See Table 3 for more details.

**Table 3:** Comparison of Teaching Quality between the Two Groups (%, n)

Group	Number of cases	Improve learning interest	Improve problem-solving ability	Improve clinical thinking ability	improve autonomous learning ability
Control group	28	20 (71.43%)	20 (71.43%)	19 (67.86%)	18 (64.29%)
Observation group	28	27 (96.43%)	26 (92.86%)	26 (92.86%)	25 (89.29%)

χ^2^		6.487	4.383	5.543	4.909
P value		0.011	0.036	0.019	0.027

### Evaluation of students of the two groups on teaching model

There was no difference in the scores of teaching attitude and teaching content between the two groups (P > 0.05); the scores of teaching method and teaching effect in the observation group were significantly higher than those in the control group (P < 0.05), and the total score in the observation group was significantly higher than that in the control group (93.73 points vs 88.45 points, P < 0.05). See Table 4 for details.

**Table 4:** Comparison of Evaluation Results of the Two Groups on Teaching Model (points, x̅ ± s)

Group	Number of cases	Instructor attitude	Guidance teaching content	Guidance teaching method	Guidance teaching effect	Total score
Control group	28	18.91±0.76	38.34±1.73	23.64±2.11	7.65±0.71	88.45±2.64
Observation group	28	19.02±0.69	38.77±1.90	28.21±1.93	8.43±0.56	93.73±2.34

t value		-0.567	-0.885	-8.457	-4.564	-7.920
P value		0.573	0.380	0.000	0.000	0.000

### Satisfaction with teaching between the two groups

The students' satisfaction with teaching in the observation group was 96.43%, which was significantly higher than that in control group (78.57%, P < 0.05). See Table 5 for details.

**Table 5:** Comparison of Satisfaction with Teaching between the Two Groups (%, n)

Group	Number of cases	Very satisfied	Satisfied	Basically Satisfied	Dissatisfied	Generally satisfied
Control group	28	4	6	12	6	22 (78.57%)
Observation group	28	13	9	5	1	27 (96.43%)

χ^2^						4.028
P value						0.043

## Discussion

Breast disease is a serious threat to women's life health and safety. It is very important for hospitals to train professional breast specialists 9,10. The traditional teaching model takes the teacher as the dominant and takes the theory teaching as the focal point to carry on the filling-in teaching, and the student receives the spoon-feed and strongly relies on the teacher. Although it can make the interns master the breast related professional theory knowledge, the students lack the independent thinking ability 11, lacking of ability in the processing information and the pioneering thinking, with poor effect in operation hands-on ability training. In the course of clinical practice, hands-on practice ability is often out of the basic theory, interns' independent ability is difficult to be exercised, and due to the particularity of breast, patients are more sensitive to the examination of breast. This demonstration teaching has always been a difficult problem in the traditional teaching, plus the limited clinical level of some teachers, it is impossible to accurately show the method of breast examination to the students 12, and therefore, exploring new and more effective teaching methods is helpful to improve students' learning and thinking ability and hands-on ability, and it is also very important to improve teaching effect.

In 1969, Barrows HS, a professor of neurology in the United States, founded the problem-based teaching method, PBL, at McMaster University, Canada, which is a teaching method that inspires students' motivation to learn and guides them to master the content of their studies with clinical problems 13,14. At present, it has become one of the most widely used clinical teaching models at home and abroad 15,16. Its essence is to arouse the students' learning interest with the problems and actual cases, and instructors guide side by side and make the students change from the traditional passive learning to the active learning mode, which enhances the students' ability of autonomous learning to a great extent 17. However, multi-media teaching is characterized by large amount of information, colourful content, strong expressive power and easy to operate, which can reduce the teaching difficulty of teachers and make the classroom atmosphere more interesting, and it is the most indispensable teaching tool for teachers 18,19.

For the multi-media combined PBL teaching model used in this study, through the improvement of traditional teaching model, students are encouraged to participate in various teaching links, so that students can change from passive learning to active learning, and on the basis of improving learning efficiency, students can cultivate their active learning habits and problem-finding and problem-solving ability, and then through playing pictures to students and combining multi-media animation demonstration, video and audio data, abstract medicine is presented in an intuitive and vivid way, and inserted into clinical teaching so as to make students have profound perceptual understanding and improve the teaching level of breast surgery.

The results showed that the total scores of breast examination, differential diagnosis, imaging reading, diagnosis and treatment scheme formulation, and theoretical knowledge in the observation group were higher than those in the control group (P < 0.05). The total scores of body position and surgical site selection, disinfection and local anesthesia, surgical operation, postoperative treatment and clinical operation skills in the observation group were also higher than those in the control group (P < 0.05).

This is because PBL teaching can be extended according to the evolution of the patient's condition and the diagnosis and treatment process. At the same time, with the help of multi-media data, students can quickly enter the role of doctors, teachers can cleverly design tips, and apply multi-media teaching method to help students understand relevant knowledge in an all-round and in-depth way, and make students have the opportunity to master standard clinical operation, not only vivid, but also expanding the teaching content of breast surgery, laying a solid foundation for clinical practice 20.

At the same time, the students' evaluation and satisfaction of the multi-media combined PBL teaching model are high, which can improve their learning interest, problem-solving ability, clinical thinking and self-learning ability (P < 0.05), which indicates that the multi-media combined PBL teaching method is effective in the teaching of breast surgery.

In conclusion, multi-media combined PBL teaching can effectively improve students' professional skills, enhance students' enthusiasm and initiative, and has high teaching satisfaction among students, thus deserving to be popularized in the clinical teaching of breast surgery.
